# Microstructure and Wear Resistance of Ti6Al4V Titanium Alloy Laser-Clad Ni60/WC Composite Coating

**DOI:** 10.3390/ma17010264

**Published:** 2024-01-04

**Authors:** Mingjia Feng, Yunhai Ma, Yitong Tian, Hongtu Cao

**Affiliations:** 1 The College of Biological and Agricultural Engineering, Jilin University, 5988 Renmin Street, Changchun 130025, China; 2 School of Mechanical and Civil Engineering, Jilin Agricultural Science and Technology University, Jilin 132101, China; 3 Key Laboratory of Bionic Engineering, Ministry of Education, Jilin University, 5988 Renmin Street, Changchun 130025, China

**Keywords:** laser cladding, Ni60/WC, scanning speed, titanium alloys, wear behavior

## Abstract

In this paper, Ni60/WC wear-resistant coatings have been created on the Ti6Al4V substrate surface using a pre-layered powder laser cladding method by deploying various scanning speeds of 8, 10, 12, and 14 mm/s. The coatings are characterized through X-ray diffraction (XRD), scanning electron microscopy (SEM), and a high-speed reciprocating fatigue wear tester. It is found that the phase composition of the coating comprises the synthesized, hard phase TiC and TiB_2_, the silicides WSi_2_ and W5Si_3_, and NiTi and γ-Ni solid solutions. At different scanning speeds, there is a metallurgical fusion line in the bonding area of the fused cladding layer, indicating a good metallurgical bonding between the substrate and the powder. At a low scanning speed, the coating develops into coarse dendrites, which shows significant improvement with scanning speed. The microhardness first increases and then decreases with the scanning speed, and the coating’s average microhardness was 2.75–3.13 times higher than that of the substrate. The amount of mass wear has been reduced by 60.1–79.7% compared to the substrate. The wear behavior of the coatings was studied through detailed analysis of wear surfaces’ microstructures and the amount of wear to identify the optimum scanning speed.

## 1. Introduction

Titanium alloys, well-known for their advantages of high strength yet low density, good mechanical properties, excellent toughness, and corrosion resistance, have found widespread applications in aerospace, petrochemical, automotive, and biomedical industries in recent decades [1,2,3,4]. As a biomaterial, titanium alloys have better biocompatibility and corrosion resistance than magnesium alloys [5,6,7]. However, their utility in demanding wear environments, such as in aircraft engine pressurizers and structural components of rockets, missiles, and high-speed aircraft, is hindered by inherent issues like low hardness, susceptibility to adhesion, inadequate wear resistance, and poor oxidation resistance at elevated temperatures [8,9,10]. In order to improve its wear performance while maintaining the overall excellent performance of the material itself, the use of a surface modification technique has been acknowledged as an effective approach for improving the wear resistance by the majority of scholars [11].

As a new type of surface modification technology, the laser cladding technique shows superior advantages in terms of high energy density, rapid cooling speed, and low dilution rate. It enables the formation of coatings with good metallurgical bonding and excellent thickness controllability [12,13,14,15,16]. This method can combine the significant toughness of the matrix material with the considerable hardness and wear resistance of the surface coating, thus significantly improving the overall performance of the material. Wang et al. [17] used a coaxial powder feeding device to laser clad Ni60/SiC composites on a Ti-6Al-4V substrate. The hardness of composite coatings reached 2–3 times that of the substrate material with significantly improved wear resistance. The optimal combination of material compositions has been identified as Ni60 and SiC powders. Yan et al. [18] deployed laser melting to in situ synthesize a Ti_3_SiC_2_ phase and silicide-reinforced, Ni-based composite to fabricate self-lubricating coatings on Ti-6Al-4V substrate with a Ti-Si-C system and NiCrBSi powder as raw materials. The fabricated coatings have the characteristics of minimal abrasion and plastic deformation. Jiang et al. [19] used the WC-17Co composite as a laser melting material to study the influence of laser powers on the hardness, organization, and wear behavior on the coating’s surface. The results showed that the surface created with a laser power of 2000 W showed superior hardness and wear resistance.

In view of the results of previous studies, it is known that the use of laser cladding technology can indeed improve the surface properties of titanium alloys. But, the cladding material is an important factor affecting the quality of the coating. The high brittleness of SiC materials can make the coatings prone to cracks and other defects. And, there is a lack of systematic research on the effect of laser scanning speed on the microstructure and wear resistance of the coating. The use of materials with similar coefficients of thermal expansion is an effective way to reduce the generation of the above defects. Tungsten carbide is a ceramic material with ultra-high hardness, and its coefficient of thermal expansion is similar to that of titanium alloys. WC Ni-based alloys have good wettability, and the use of laser-melted, WC, hard-particles-enhanced, Ni-based alloy powder coating was found to improve the wear resistance of the substrate [20,21,22,23]. It is worth noting that there are few studies reporting the production of composite coatings on titanium alloy surfaces through laser melting of Ni60 + 35% WC powder. There is a lack of systematic studies on the effect of the laser scanning speed on the microstructure and wear resistance of Ni60 + 35% WC composite coatings.

**Figure 1:**
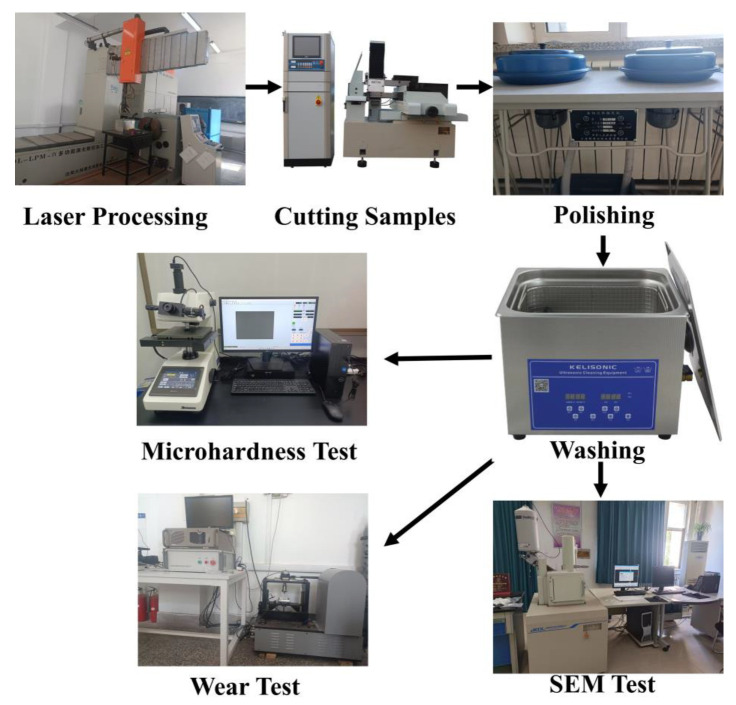
Process flow diagram.

In this paper, Ni60 + 35% WC powder was used to prepare coatings on Ti6Al4V substrate with different laser scanning speeds. Under the scanning speeds of 8, 10, 12, and 14 mm/s, the effects of phase composition, microstructure, microhardness, friction, and wear on the surface properties of the coatings were explored. The wear behavior and mechanism were investigated by analyzing the wear surfaces and the amount of wear to identify the optimal scanning speed. The process flow diagram of this paper is shown in Figure 1. This study can extend the application range of titanium alloy under severe wear conditions. It can improve the service life and reduce the cost of titanium alloy used as a pressurizer in aircraft engines. It also has a certain reference value and theoretical basis for the reasonable selection of the laser scanning speed and the preparation of wear-resistant coatings of titanium alloy.

## 2. Materials and Characterization

### 2.1. Materials

**Figure 2:**
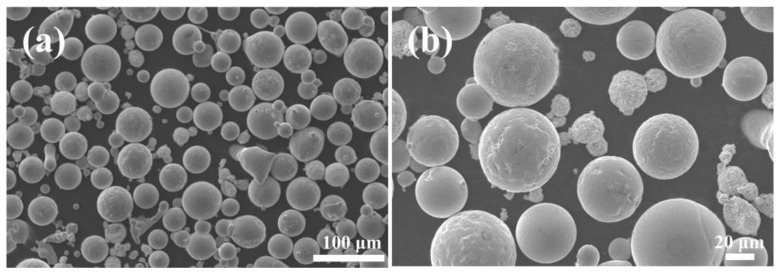
Morphology of Ni60–35WC hybrid powder at different magnifications. (**a**) Microstructure at low magnification (**b**) Microstructure at high magnification.

A Ti6Al4V alloy plate in a hot-rolled state with a size of 50 mm × 50 mm × 5 mm was used as the substrate in this paper. Before the laser fusion cladding process, the surface oxide layer was removed by grinding with sandpaper, and then it was ultrasonically cleaned with acetone. The fusion cladding material comprised a blend of Ni60 (45–100 μm) and WC (45–150 μm, 99.9% purity) powders, with a 35% weight fraction of WC in the mixed powders. Mixed powder was purchased from Nangong Hangzhuo Metal Products Co., Ltd. (Nangong, China). The chemical compositions of the substrate Ti6Al4V alloy plate as well as the Ni60 powder are described in Table 1 and Table 2. Figure 2 shows the morphology of the pre-placed mixed powders at different magnifications.

### 2.2. Laser Cladding and Sample Preparation 

**Figure 3:**
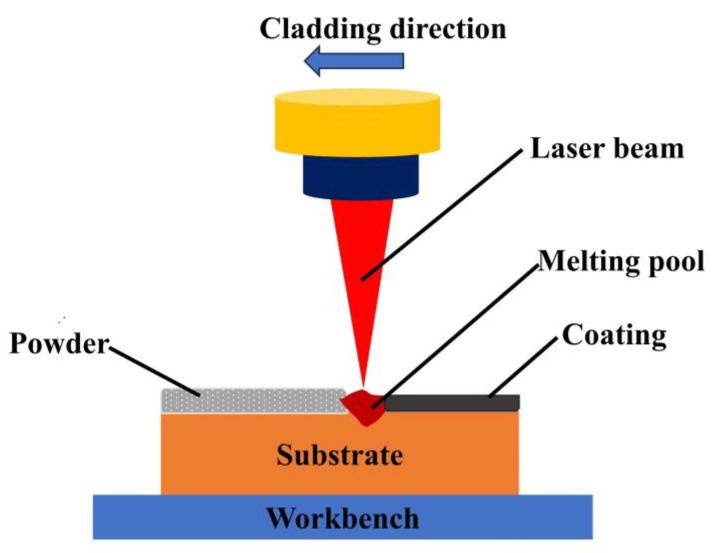
Schematic diagram of laser cladding processing.

The presetting powder method was adopted before the laser cladding experiment, which involves the application of a uniform presetting powder with a thickness of 1 mm on the Ti6Al4V substrate using an organic binder. The powders were placed in a drying stove and dried at 100 °C for 2 h before the experiments. A DL-HL-T200 cross-flow CO_2_ laser (Shenyang Continental Laser Complete Equipment Co., Ltd., Shenyang, China) was deployed in the experiment, which has a maximum power of 2000 W. Schematic diagram of laser cladding processing can be seen in Figure 3. The experimental parameters are shown in Table 3. The spot diameter is 3 mm, the lap rate is 30%, and argon is used as the protective gas for the cladding process (flow rate 5 L/min). The laser cladding process was performed at room temperature, and specimens were naturally cooled in air after cladding. The metallographic and wear specimens were processed into 15 × 10 × 5 mm samples for grinding and polishing, and the metallographic samples were corroded with HF:HNO_3_:H_2_O at a volume ratio of 4:10:86 as a corrosive solution for 30 s. Then, the samples were dried using a drying stove.

### 2.3. Microstructure Inspection and Performance Testing 

**Figure 4:**
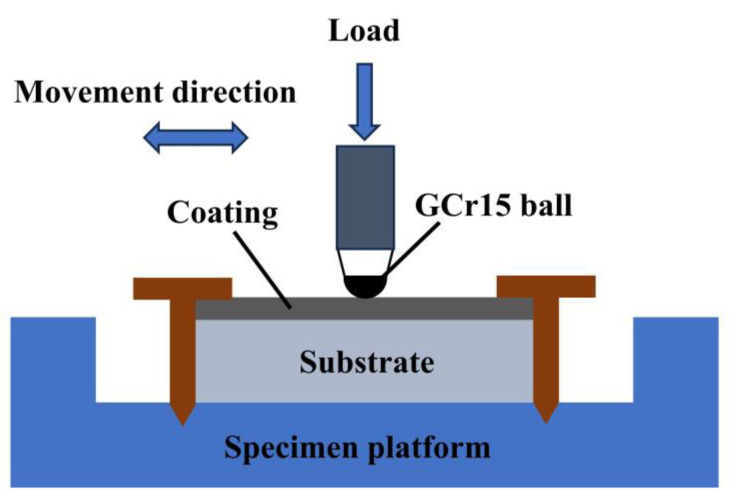
Schematic diagram of friction wear test.

The microstructure of the fused cladding layer was examined using a LEICA DM2700M (Leica Instruments GmbH, Wetzler, Germany) optical microscope (OM) and a JSM-6490LV (JEOL Ltd., Akishima City, Tokyo, Japan) electron microscope (SEM). The elemental composition and content of the constituents in the fused cladding layer were determined through energy-dispersive spectroscopy (EDS) coupled with SEM. The physical phase analysis of the cladding was conducted using a Shimadzu XRD-6100 (Shimadzu Kyoto, Japan) X-ray diffractometer (XRD) with a Cu target, a tube voltage of 40 kV, a tube current of 300 mA, a scanning speed of 4°/min, a scanning angle step of 0.02°, and a diffraction angle scanning range of 20° to 90°. Microhardness testing was performed using an MH-500 digital microhardness tester with a 500 gf load and a 10 s loading time. To minimize measurement variability, hardness was tested at five positions at the same height below the coating surface, and the average value was recorded. Friction and wear tests were conducted at room temperature using an MGW-02 high-speed reciprocating fatigue friction and wear tester. The schematic diagram of the friction and wear setup is shown in Figure 4. GCr15 material with a diameter of 6.5 mm served as the grinding ball, and a loading force of 40 N was applied with an oscillation frequency of 5 Hz. The experiments were carried out for 20 min. 

## 3. Results and Discussion

### 3.1. Phase Composition and Microstructure 

**Figure 5:**
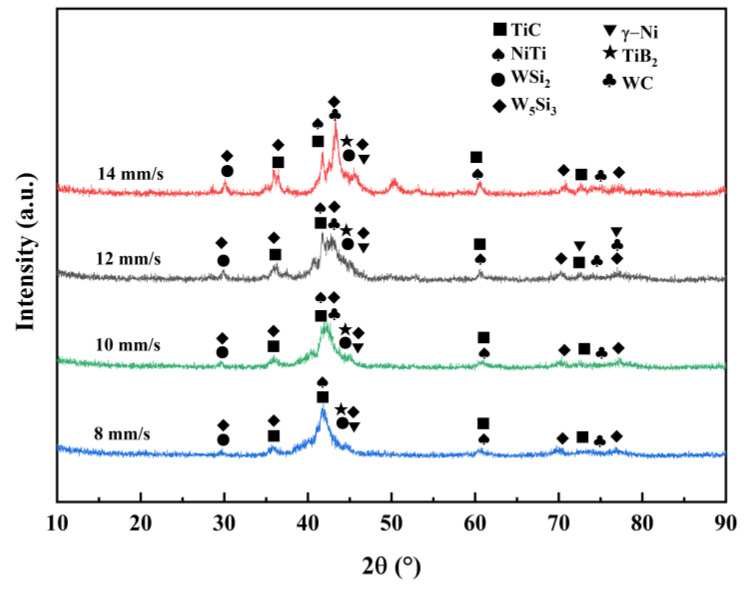
XRD pattern of laser-melted Ni60–WC composite coating.

To analyze the physical phase composition of Ni60/WC composite coatings, XRD was used, and Figure 5 shows the XRD patterns of Ni60/WC-composite-coated surfaces at various speeds (8, 10, 12, and 14 mm/s). The composition of physical phases is similar at different scanning speeds. The physical phase primarily consists of hard phases TiC, TiB_2_, WC, and γ-Ni solid solution, along with intermetallic compounds NiTi, WSi_2_, and W_5_Si_3_. Owing to the elevated temperature in the melt pool, some of the WC can undergo dissolution, leading to matrix dilution within the melt pool. This results in the reaction within the melt pool to produce TiB_2_, NiTi, TiC, WSi_2_, W_5_Si_3_, and other phases [24]. XRD results demonstrated that the diffraction peaks of TiC and NiTi are stronger, indicating that TiC and NiTi are produced in the composite coating. Simultaneously, the diffraction peaks of WC can be observed, indicating that some of the composite coating still remains as dissolved WC particles.

The XRD results prove that the hard phase is distributed within the composite coating and accompanied by the presence of compounds, such as silicides. The study of chemical reactions in the melt pool is of great significance, as it helps with the accurate prediction of possible reactions in the melt pool and facilitates controlling the organization and relevant properties of the coating. During laser melting, the matrix Ti6Al4V and Ni60/WC powders undergo complex physicochemical reactions to form different phase compositions during the melt pool reaction [25]. Firstly, under the intense energy of the laser beam, Ni60 powder undergoes dissolution, and WC particles experience partial dissolution. Simultaneously, the matrix Ti6Al4V is diluted, and elements, such as Ti, Ni, B, Si, C, and W, may be present in the molten pool. The chemical reactions are:

Ti + C → TiC(1)

Ti + 2B → TiB_2_ (2)

Ni + Ti → NiTi (3)

W + 2Si → WSi_2_ (4)

5W + 3Si → W_5_Si_3_ (5)

**Figure 6:**
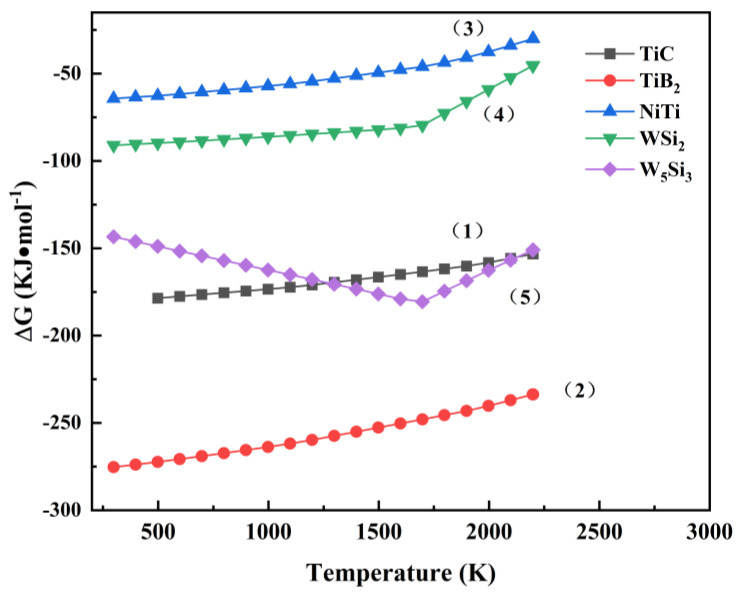
Variation of standard ΔG with temperature for reactions (1)–(5).

Based on the principles of thermodynamics, a negative change of Gibbs free energy (ΔG) suggests the chemical reaction could be completed spontaneously under that condition [26]. Figure 6 shows the Gibbs free energy–temperature curves, and the standard Gibbs free energies ΔG of TiC, TiB_2_, NiTi, W_5_Si_3_, and WSi_2_ are consistently below zero within the temperature range of 298~2200 K. This is similar to the results reported in the literature [27,28], which indicates that all of the above substances can be synthesized during the laser cladding process [29,30].

**Figure 7:**
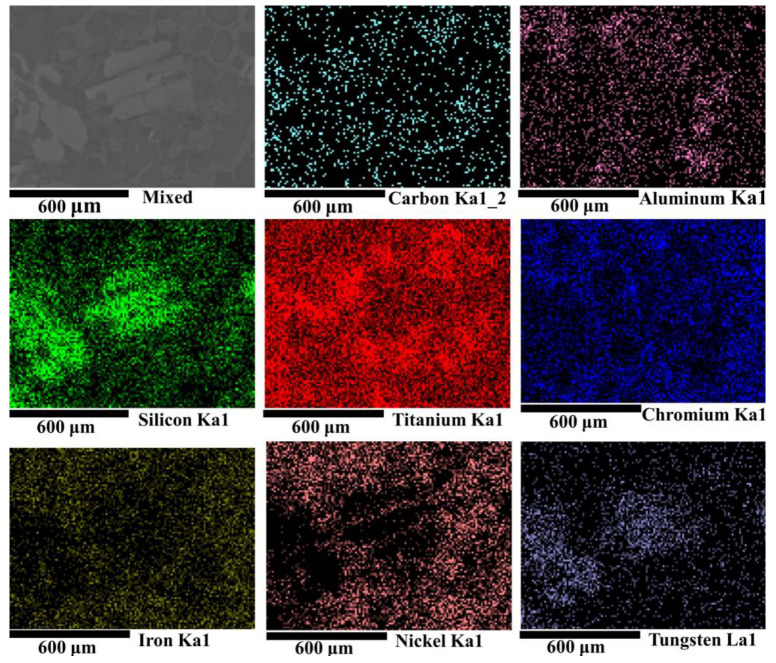
EDS surface scanning results of Ni60/WC composite coating with a scanning speed of 12 mm/s.

Figure 7 depicts the surface scanning results of the microstructure of the composite coating at a scanning speed of 12 mm/s. It can be observed that the large grayish-white irregular precipitates consist of elements W and Si, which can be identified as the compounds of WSi_2_ and W_5_Si_3_. Based on the Gibbs free energy and thermodynamic theories discussed earlier, it is easier to produce W_5_Si_3_ during equilibrium solidification, followed by the formation of WSi_2_. The superior hardness and unique chemistry of WSi_2_ and W_5_Si_3_ contribute to the enhanced hardness properties and improved wear resistance [31]. Apart from large gray-white irregular precipitates, the remaining areas primarily consist of Ni, Fe, Ti, and other elements, forming the γ-Ni solid solution. The distribution of Ti elements indicates their incorporation during laser melting.

**Figure 8:**
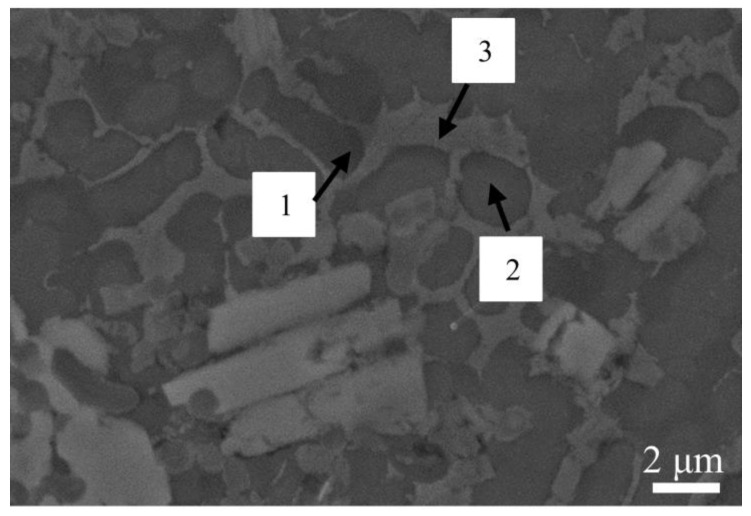
SEM image of the intermediate coating with a scanning speed of 12 mm/s.

As shown in SEM images obtained with a scanning speed of 12 mm/s in Figure 8, large columnar and massive gray-white crystalline precipitates can be observed, which are primarily composed of the elements W and Si. This indicates the presence of the compounds WSi_2_ and W_5_Si_3_. The remaining is mainly composed of gray continuous matrix and bar-like and polygonal tissues. EDS analysis was performed on microregions 1, 2, and 3, as labeled in the figure, and the atomic percent content obtained is detailed in Table 4. In microregion 1, the dark gray matrix is rich with C, Ni, and Ti elements. These three microregions are mainly rich in C, Ni, and Ti elements, and the combination of XRD results shows that the three microregions are judged to be composed of TiC, NiTi, and r-Ni solid solutions.

**Figure 9:**
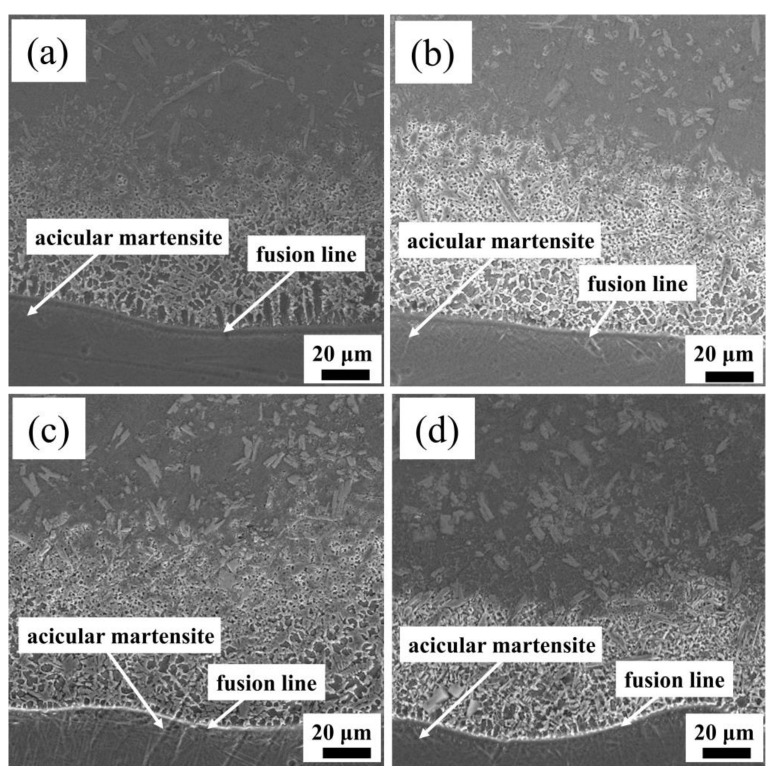
SEM images of Ni60/WC coating bonding areas at (**a**) 8 mm/s; (**b**) 10 mm/s; (**c**) 12 mm/s; (**d**) 14 mm/s.

The SEM images of the binding area of the Ni60/WC composite coating can be seen in Figure 9. A fusion line is evident at the junction of the bottom of the coating and the substrate. When the scanning speed increases, the fusion line becomes clearer and more distinct, indicating a robust bonding between the coating and the substrate [32]. The substrate and the powder diffuse with each other when affected by the action of the high-energy of laser beam, facilitating the formation of a strong metallurgical bond. Acicular martensite also appeared in the heat-affected zone at the bottom of the coating. This formation is attributed to the intensive heat generated by the high laser energy beam, resulting in rapid heating of the contact area with the substrate and a high cooling rate of 200 °C/s. The rapid cooling prompts the diffusion-free martensite transformation [33]. The acicular martensite growth direction is perpendicular to the fusion line because the optimal growth direction of crystal grains is in the same direction as the direction of heat dissipation, a phenomenon similar to the results of Zhu et al. [34]. It can be seen from the figure that there is a good metallurgical bonding between the powder and the substrate at different scanning speeds, and no obvious cracks are produced.

**Figure 10:**
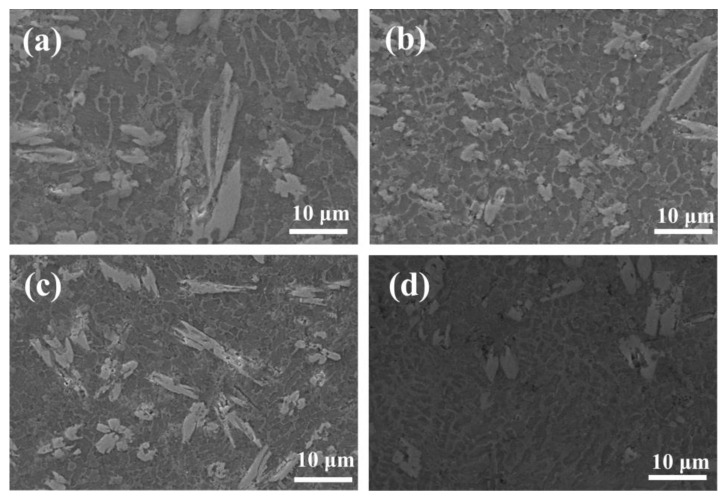
SEM images of a dendrite microstructure in the middle region of the Ni60/WC coating at (**a**) 8 mm/s; (**b**) 10 mm/s; (**c**) 12 mm/s; (**d**) 14 mm/s.

Figure 10 shows a SEM image of a microstructure in the middle region of these composite coatings at various scanning speeds. At a scanning speed of 8–10 mm/s, the low scanning speed results in the powder absorbing more laser energy per unit time. This leads to an extended heating time in the molten pool and elevation of the molten pool temperature with slow heat dissipation. Additionally, the extended heating time allows for the formation of a coarse dendrite in the fused cladding layer, hence reducing the cladding layer’s toughness value. With higher speeds, the powderless laser energy was absorbed within a specific unit time, thus shortening the heating time in the molten pool, reducing the molten pool temperature, and accelerating the cooling process. This results in the formation of a fine and dense microstructure with more nuclei in the fused cladding layer, which improves the hardness of the coating. Notably, at a scanning speed of 12 mm/s, the microstructure exhibits a greater density of fine dendrite. 

### 3.2. Microhardness

**Figure 11:**
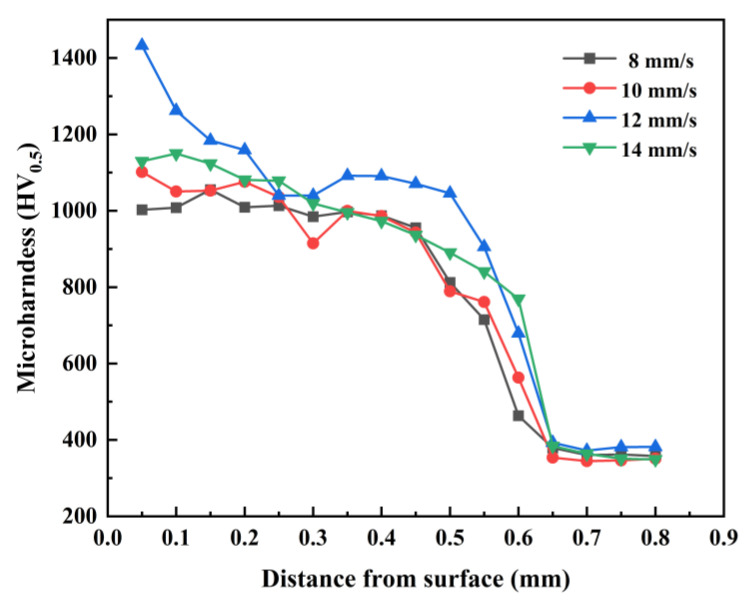
Microhardness of fused coatings with different scanning speeds.

The microhardness of the fused-coated samples from the top to the substrate fabricated at various scanning speeds is depicted in Figure 11. It is evident that the microhardness of the fused-coated layer presents a higher value compared to the substrate (364.8 HV_0.5_). In the heat-affected zone at the interface, the microhardness of the coating experiences a drastic drop. Despite the generation of an acicular martensitic phase in the heat-affected zone, the microhardness is still higher than that of the matrix. The decrease in microhardness near the coating/substrate interface is greater due to dilution of the bottom of the coating by the substrate material. Meanwhile, the microhardness of the substrate area remains hardly changed. The average hardness of the fused cladding layer at scanning speeds of 8, 10, 12, and 14 mm/s were 1001.4 HV_0.5_, 1017.8 HV_0.5_, 1141.6 HV_0.5_, and 1037.7 HV_0.5_, respectively, indicating a 2.75–3.13 times increase compared to that of the substrate. Liu et al. [20] prepared a Ni60 + 50% WC coating on a Ti6Al4V substrate, and the hardness increased by 2.81 times. Feng et al. [35] prepared a (Ti3Al + TiB)/Ti composite coating, and the hardness was 2.31 times that of the substrate, which is lower than the hardness of the fused cladding layer in this paper. The microhardness of the fused-coated samples first increased and then decreased with the rise of the scanning speed, and the highest average hardness of the fused-coated layer was achieved when the scanning speed was set at 12 mm/s.

The hardness of the cladding layer is significantly higher than that of the substrate due to the TiC, TiB_2_, W_5_Si_3_, and WSi_2_ hard phases generated in the composite coating, which is conducive to the improvement of the microhardness of the coating. The high cooling rate and the presence of silicides in the laser cladding inhibit grain growth [36], thus leading to grain refinement and strengthening. A tiny grain size means that there are more grain boundaries in the composite coating, which prevents the movement of dislocations and makes the composite coating less susceptible to plastic deformation. The tissue of the coating at a 12 mm/s scanning speed is finer and denser than the rest of the coatings, and hence the average microhardness of the coating is highest at this speed.

### 3.3. Friction and Wear Performance

**Figure 12:**
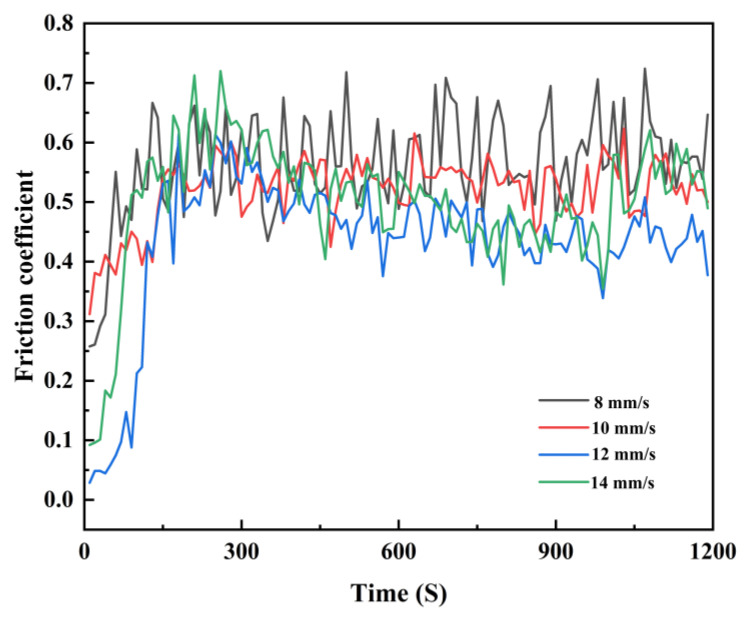
Coating friction coefficient at various scanning speeds.

The friction coefficients of the fused coatings under various scanning speeds are presented in Figure 12. The average friction coefficients of the Ni60/WC coatings exhibit a progressive trend, measuring 0.56, 0.52, 0.43, and 0.5 as the scanning speed increases from 8 mm/s to 14 mm/s. Mthisi et al. [37,38] prepared different composite coatings on the substrate with friction coefficients ranging from 0.6–0.76; the results of this paper are similar to theirs and have lower coating friction coefficients. This pattern demonstrates an initial decrease followed by an increase in friction coefficients with the rising scanning speed, which aligns with the observed variations in microhardness. Notably, the microhardness of the coating peaks at 12 mm/s, thus enhancing its resistance to abrasive forces and leading to improved wear resistance and a smaller friction coefficient. During the initial friction stage, the sample surface appears rough, and the friction coefficient undergoes significant fluctuations. In the stable friction stage, the friction coefficient of the coating exhibits fluctuations within a certain range, which are possibly attributed to the micro-cutting of hard-phase particles within the coating upon contact with the counter-abrasive ball [39]. 

**Figure 13:**
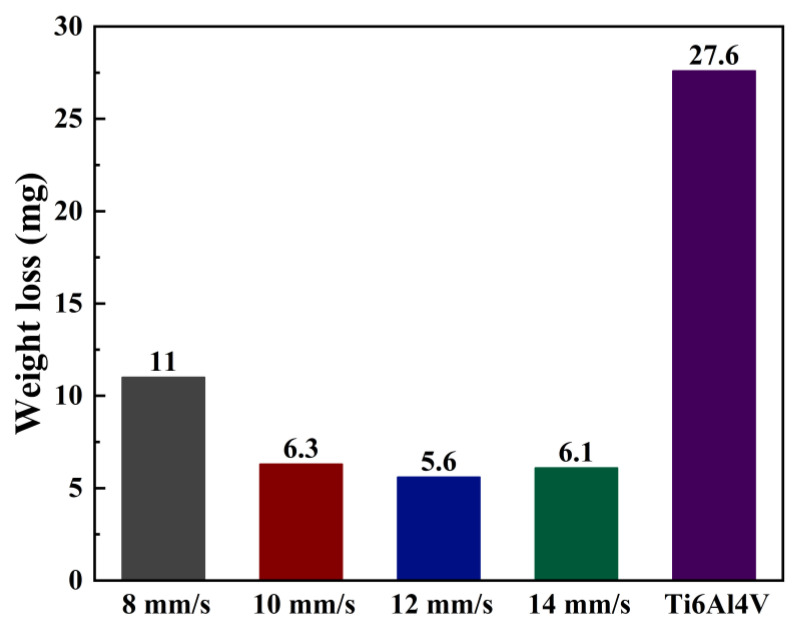
Coating and substrate wear weight loss plots at various scanning speeds.

Figure 13 depicts the histograms of wear weight loss of fused samples and substrate Ti_6_Al_4_V at various scanning speeds The wear weight first decreases and then increases. The weight loss of the coating is significantly smaller compared to that of the substrate. This is consistent with the variation trend of the friction coefficient. The greater hardness of the material contributes to better wear resistance [40]. As the speed changes from 8 to 14 mm/s, the Ni60/WC wear weight loss can be reduced by 60.1%, 77.2%, 79.7%, and 77.9%, respectively, compared to that of the substrate. This results in a maximum increase of 4.9 times. The observed improvement in wear resistance of the laser-fused cladding relative to substrate Ti_6_Al_4_V surpasses that in the literature [41], where TiC/Ni60 coatings on the substrate Ti_6_Al_4_V achieved a 4-fold improvement in wear resistance, which is less than the 4.9-fold improvement observed in the coatings developed in this study. The impressive wear resistance exhibited by the fused samples in room temperature, dry sliding friction tests can be attributed to the reaction of Ni60/WC powders within the laser melt pool. This reaction leads to the formation of the corresponding hard phases, thus significantly enhancing the wear resistance of the fused samples [42]. These mechanical property results indicate that laser cladding Ni60 + 35% WC is an effective method to improve the surface mechanical properties of titanium alloys.

**Figure 14:**
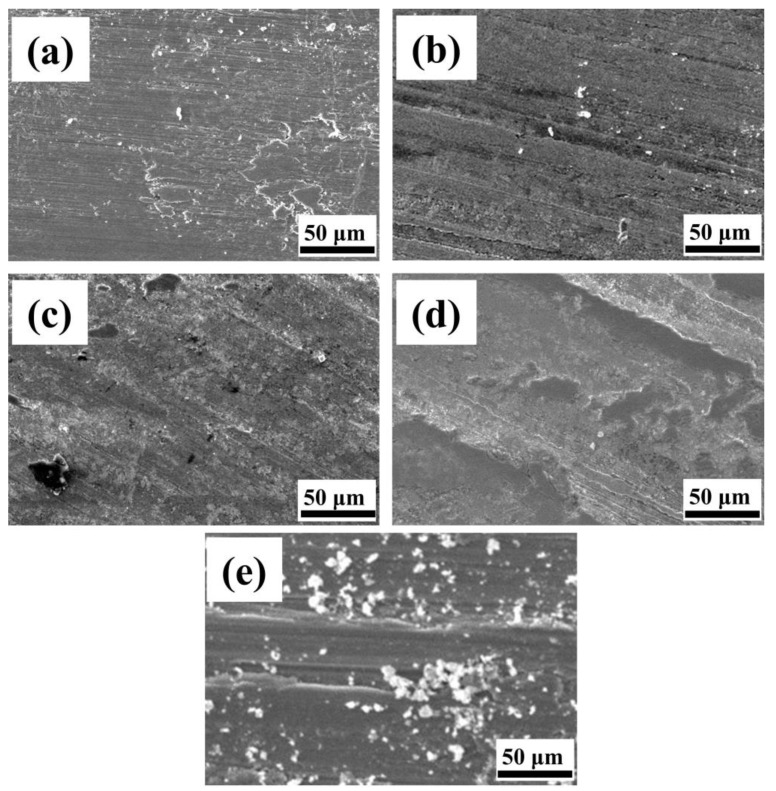
Wear surfaces of Ni60/WC coating with Ti6Al4V substrate. (**a**) Scanning speed 8 mm/s coating; (**b**) Scanning speed 10 mm/s coating; (**c**) Scanning speed 12 mm/s coating; (**d**) Scanning speed 14 mm/s coating; (**e**) Ti6Al4V substrate.

The wear morphology of the substrate Ti6Al4V with the Ni60/WC fused cladding layer after dry sliding friction wear is observed and analyzed through SEM, as depicted in Figure 14. The substrate wear surface exhibits roughness with numerous abrasive grains, as well as deeper furrows and plastic deformation along the wear direction. This roughness results from the substrate surface being subjected to violent cutting action, causing more furrows and chips, and exhibiting typical abrasive and adhesive wear [43].

Compared to the wear morphology on the substrate, the wear behavior of the fused cladding specimen is characterized by micro-cutting, presenting a smoother wear surface with fewer abrasive grains on the surface. When the scanning speed is 8 mm/s and 10 mm/s, there are some furrows and a limited number of abrasive grains on the surface wear morphology, with the predominant forms of wear being abrasive wear and localized adhesive wear. This phenomenon is mainly due to the fact that the material on the surface of the fusion cladding layer flakes off to form abrasive chips and enters the friction substrate to form abrasive grains, thus contributing to the formation of abrasive wear and furrow formation. When the speed increases to 12 mm/s and 14 mm/s, the amount of grooves seen decreases, and there is no debris on the wear surface. A grayish-white hard phase is uniformly and tightly embedded in the coating surface. During wear, the hard phases, which get embedded onto the surface, can resist the external load on the friction side, resulting in shallower grooves on the wear surface. The increased presence of hard phases can weaken the cutting action during grinding, thus causing shallower grooves and minimal abrasive wear. The abundance of hard phases significantly improves the wear properties during friction wear experiment testing [44].

### 3.4. Coating Wear Mechanism 

**Figure 15:**
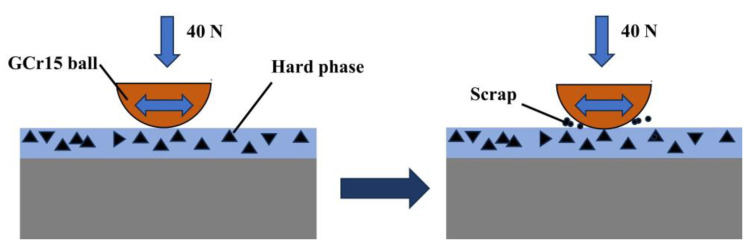
Schematic diagram of frictional wear of Ni60/WC composite coating.

The friction and wear model of the Ni60/WC coating is given in Figure 15. The cladding process generates a substantial amount of heat, which causes part of the WC particles to dissolve and the substrate to dilute into the molten pool. This initiates reactions that produce TiC and TiB_2_ hard phases and W_5_Si_3_ and WSi_2_ silicides. These hard phases improve the hardness of the substrate, while the silicide can improve its toughness, thereby contributing to improved wear resistance. During the initial stage of the wear experiment, when the pair of grinding balls contacts the smooth coating surface, the friction coefficient is small. However, with an increase in friction time, the hard phases (TiC and TiB_2_) as well as the W_5_Si_3_ and WSi_2_ silicides and the WC particles inside the coating become exposed on the surface. These elements come into contact with the grinding ball, causing a cutting phenomenon and resulting in an obvious rise of the friction coefficient. As the wear between the ball and hard-phase particles increases, the hard-phase particles are fractured, resulting in the formation of abrasive debris and abrasive wear of the coating. Once the friction time accumulates to a certain point, the peaks of the fabricated coating surface smooth out, thus raising the surface’s contact area. Its wear process enters a stable stage, and the friction coefficient is stabilized with minimal fluctuation.

The microhardness of the GCr15 pair of grinding balls is 800 HV [45], which is much higher than the microhardness of the Ti6Al4V substrate (about 364.8 HV). Therefore, the convex body on the surface of the counter-abrasive ball easily passes through the sliding surface of the substrate, and the wear surface of the Ti6Al4V substrate shows deeper plough furrows, adhesion, and wear debris characteristics, as shown in Figure 14e. The Ti6Al4V substrate produces plastic deformation, and the wear mechanism of the substrate is abrasive wear and adhesive wear.

The TiC, NiTi, and TiB_2_ reinforcing phases play a “skeleton” support in the composite coating, while the silicides W_5_Si_3_ and WSi_2_ have a high hardness and strong covalent atomic bonds, which can effectively prevent the abrasive particles from embedding in the surface to prevent the coating from further wear and deformation. From Figure 14a–d, it can be seen that the wear surface of the coating is relatively smooth, with a small number of shallow furrows. The wear mechanism of the composite coating is mainly abrasive wear.

The amount of wear features one of the fundamental parameters reflecting the wear resistance of a material [46]. Based on Archard’s formula [47], the amount of wear is inversely proportional to the material’s hardness. Consequently, higher hardness implies better abrasion resistance. This result is like the findings of Mu et al. [48], who concluded that a lower coefficient of friction and higher surface microhardness can improve wear resistance. In this study, Figure 10 indicates that the microhardness of the composite coating is considerably improved when compared to the substrate material, with the highest microhardness achieved when 12 mm/s was set as the scanning speed. This implies that the wear resistance of the coating is superior to that of the substrate.

## 4. Conclusions

This paper focuses on deploying the pre-positioned powder laser cladding technique to create composite cladding layers of Ni60 + 35% WC based on a Ti6Al4V substrate. The influences of various laser scanning speeds (8, 10, 12, and 14 mm/s) have been thoroughly explored to strengthen and optimize the tribological properties of the Ti6Al4V substrate. The key findings can be found as follows:

1. The phase compositions of the composite coatings under different scanning speeds are fundamentally similar, and are mainly composed of γ-Ni solid solution, TiC, TiB_2_, WSi_2_, W_5_Si_3_, NiTi, and residual WC. Good metallurgical bonding between the matrix and coatings is consistently observed under different scanning speeds.
2. The laser-melting Ni60/WC composite coating significantly enhances the microhardness of the Ti6Al4V substrate. The microhardness exhibited first increases and then changes to a decreasing trend with the scanning speed. When 12 mm/s was deployed as the scanning parameter, the average microhardness of the produced coating was 3.13 times higher than that of the substrate (364.8 HV_0.5_) (ca. 1141.6 HV_0.5_). This phenomenon may be owing to the fact that the harder phases and finer microstructure were produced at this scanning speed.
3. The composite coatings demonstrate excellent tribological performance under dry sliding wear test conditions compared to the Ti6Al4V alloy. Both the friction coefficient and the weight of abrasion loss show a rise and then a decline with the scanning speed, and the optimal tribological performance is achieved at a set scanning parameter of 12 mm/s.
4. The wear mechanism of the Ti6Al4V matrix is produced through a combined interaction of intense abrasive wear along with adhesive wear. In contrast, the wear of the Ni60/WC composite coating features localized adhesive wear and a slight abrasive wear mechanism. The produced coating shows the most excellent wear resistance at a scanning speed of less than 12 mm/s, which is 4.9 times higher than that of the matrix.
5. This study can broaden the use of titanium alloys in high-wear environments. It can also provide technical guidance for the preparation of anti-wear coatings and the selection of laser cladding process parameters. To further improve the surface properties of Ti6Al4V, the effect of a laser cladding bionic coating on the surface can be further explored. Some auxiliary technologies, such as ultrasonic vibration, electric field, and magnetic field, can be combined with laser cladding at the same time to explore the impact of its surface properties on substrate research.

## Figures and Tables

**Table 1:** Ti6Al4V chemical composition (wt-%).

Al	V	Fe	C	N	H	O	Ti
6.8	4.5	0.30	0.10	0.05	0.015	0.20	Bal.

**Table 2:** Ni60 powder chemical composition (wt-%).

Fe	B	Si	Cr	C	Ni
4.31	3.2	4.0055	15.9	0.78	Bal.

**Table 3:** Experimental parameters of laser cladding.

NO.	Laser Power (W)	Scanning Speed (mm/s)	Laser Beam Diameter (mm)
Sample 1	1400	8	3
Sample 2	1400	10	3
Sample 3	1400	12	3
Sample 4	1400	14	3

**Table 4:** EDS scanning results for different regions for Figure 7 above.

Region	Element Composition (at%)							
	C	Al	Si	Ti	Cr	Fe	Ni	W
1	34.700	1.351	5.487	18.330	9.798	8.371	20.907	1.056
2	23.883	-	8.037	23.418	11.230	8.600	24.832	-
3	27.695	1.613	1.632	15.715	8.498	7.790	37.056	-

## Data Availability

The data presented in this study are available on request from the corresponding author.
